# UV-durable self-cleaning coatings for autonomous driving

**DOI:** 10.1038/s41598-024-58549-y

**Published:** 2024-04-05

**Authors:** Songwei Lu, Yuejun Zhao, Edward A. P. Hellerman

**Affiliations:** Coatings Innovation Center, PPG Industries, Inc., 4325 Rosanna Drive, Allison Park, PA 15101 USA

**Keywords:** Autonomous sensor, Self-cleaning coating, UV durability, Hydrophobicity, Water contact angle, X-ray photoelectron spectroscopy, Materials science, Materials for devices, Materials for optics

## Abstract

A key technology to ensure the safety and accuracy of autonomous driving for future transportation is the cleanliness of the sensor surfaces for accurate signal reading. This study focuses on hydrophobic coatings with self-cleaning performances and UV durability, their possible degradation mechanism of static water contact angle (sWCA), and the effect of the hydrophobic surface on camera image quality. The UV-durable hydrophobic coatings are applied by a spray process followed by a thermal curing. The UV-durable hydrophobic coatings are evaluated on a vision camera under lab-simulated weathering conditions such as rain, mud, fog, and bugs, on samples as-prepared and after various hours of Weather-Ometer® weathering. The results indicate that the sWCA degradation of the UV-durable hydrophobic coatings during accelerated weathering is corresponding to the loss of fluorine (F) atomic percentage in the coatings, and the vision camera imaging quality improves significantly with the UV-durable hydrophobic coatings in comparison to an uncoated surface. The self-cleaning performances of the UV-durable hydrophobic coatings, as measured by two metrics using signal-to-noise ratio and modulation transfer function 50 loss (MTF50_loss_), linearly correlate with sWCA of the coatings. The UV-durable hydrophobic coatings on the sensor surface will significantly benefit autonomous driving specifically for accurate signal reading under inclement weather.

## Introduction

**Figure 1:**
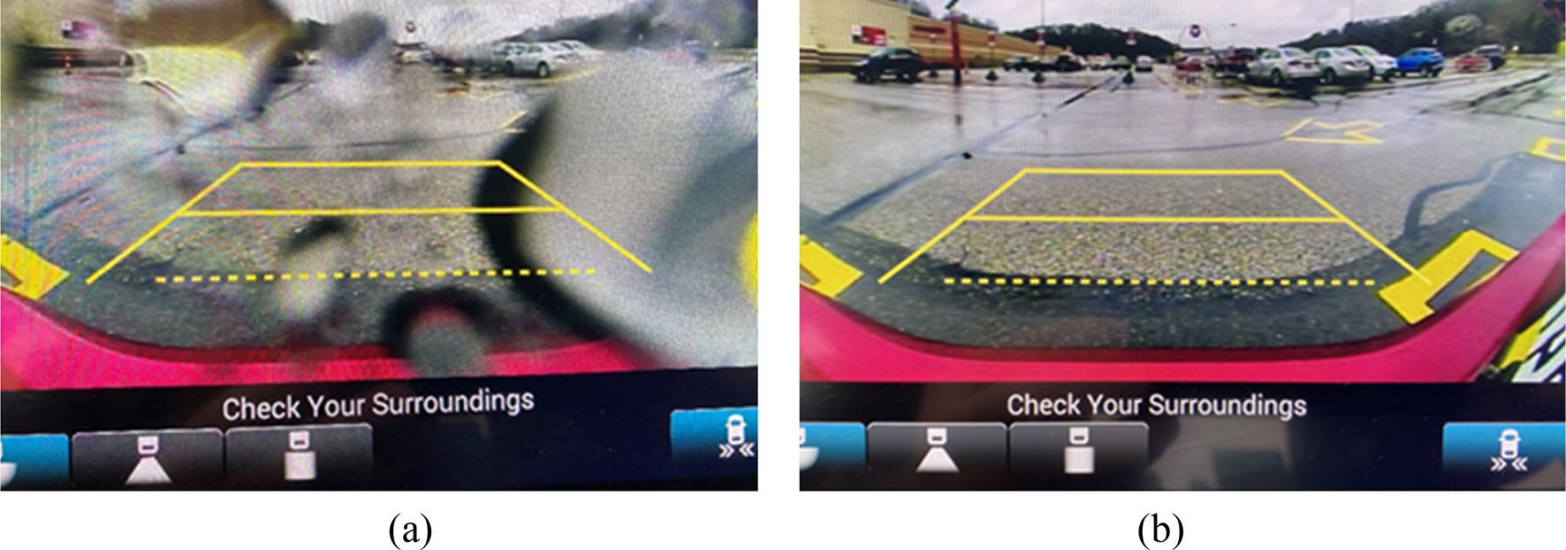
Rain droplets caused image distortion from vehicle backup camera: (**a**) distorted view with rain droplets; (**b**) clear view after cleaning rain droplets.

The emerging of future autonomous driving technologies for full automation (level 5) has the potential of evolutionally changing our society in the coming years after the recent significant adoption of Advanced Driver Assistance Systems (ADAS) including vision cameras and radars for partial automation (level 2). The future autonomous driving will reshape the entire automotive transportation, from driverless long-distance truck delivery, driverless pizza delivery, and driverless taxis, to shared vehicles. In the future, connected and automated vehicles (CAVs) will be a norm in smart cities to reduce traffic congestions and traffic accidents. However, without a driver to promptly react to a suddenly changing environment for autonomous vehicles, the sensors and the accurate information gathered from these sensors will be the critical components for autonomous vehicles to properly function. Inclement weathering, dirt on the sensor surface, and darkness will all hinder the proper functioning of the sensors. As a result, a clean and dry sensor surface will be extremely critical to mitigate dirt and/or water droplets on the sensor surface to ensure correctly gathering information for autonomous vehicles. Figure 1a was an image captured on a rainy day when rain droplets were on the surface of the backup camera of a vehicle. The image was completely distorted by the optics of the rain droplets making it challenging to clearly see other vehicles parked behind this vehicle. Figure 1b was an image taken from the same backup camera after wiping off the rain droplets. As a result of the clear image, other parked vehicles and the road condition can be seen very well.

Recently, several methods have been developed for a clean and dry sensor surface, including a piston-containing sensor cleaning device^1^, a retractable liquid-jet nozzle for spray^2^, aerodynamic shields^3^, a nozzle delivering a fluid near a center point of the sensor lens surface^4^, a hydrophilic material with a wiper on a sensor cover^5^, and a transparent ultrasonic array^6^. Clean and dry sensor surfaces are advantages of these systems, while the disadvantages include the costs of installation, refilling of cleaning solutions, and repair if a malfunction occurs. An alternative is UV-durable transparent functional coatings which have self-cleaning performances to maintain the sensor surfaces clean and dry over the time with low maintenance.

For this application of UV durable self-cleaning coatings on sensors for autonomous driving, UV durability and self-cleaning performance are two key characteristics along with coating transparency at the operational wavelength of sensors. There are many smart coatings that may be suitable for self-cleaning performances. A review of durable superhydrophobic and superamphiphobic polymeric surfaces and their applications was published by Ellinas et al. in 2017^7^. However, many of these coatings may not have UV durability, many may have limited self-cleaning performances, and some may not be transparent. Zhou et al. reported self-cleaning superhydrophobic coatings based on metal–organic frameworks (MOFs) structure with outstanding abrasion resistance and weatherability for oil–water separation and anti-corrosion application^8^. Although their superhydrophobic coatings had a high water contact angle of 169°, their weatherability testing was only limited to 500 h, which was far less than what automotive industries have demanded. Bai et al. reported a durable superhydrophobic polyester /SiO_2_-perfluorooctyltriethoxysilane coating for self-cleaning purpose using a dual-spraying method^9^. After 8 days in a UV aging test chamber, the coating has a slight degradation, but its water contact angle is still > 150°. Since the coating has a rough surface, it is unlikely the coating has high transparency with minimal haze. Wei et al. described a non-transparent polyolefin adhesive (POA)/perfluorodecyl polysiloxane modified silica superhydrophobic coating, with an initial water contact angle of around 160°^10^. The coating has excellent weatherability with the water contact angle still around 150° after 670 days of outdoor exposure. The application for presenting rain attenuation of 5G radome was evaluated with no apparent loss from the coated surface. A hydrophobic coating on glass was compared with uncoated glass for soiling effect on solar PVs in clean and polluted environments by Ratnaparkhi et al.^11^. It was found that the soiling loss was much more on the hydrophobic coated glass than on the uncoated glass. However, the authors didn’t discuss the UV durability of such hydrophobic coating coated glass, which is important for solar PV applications. The hydrophobic easy-to-clean coatings from PPG Industries, Inc. are used on computer touch screens with self-cleaning performance and excellent scratch resistance but lack significant outdoor UV durability^12^. Peczonczyk et al. investigated the fouling and cleaning of three transparent, fluorine containing functional coatings for autonomous vehicle sensors^13^. On the other hand, titania coatings have been used for self-cleaning purposes with titania’s photocatalysis performances and hydrophilicity but usually the titania coatings need several hours of UV irradiation to activate self-cleaning performances^14,15^. Fraunhofer Institute for Organic Electronics, Electron Beam and Plasma Technology recently developed a titania-based coating on thin glass as self-cleaning coatings for retrofitting facades and solar panels^16^.

In this paper, UV-durable hydrophobic (UVH) coatings will be applied on the sensor surface to assess its self-cleaning performances on a vision camera under lab-simulated weathering conditions such as rain, mud, fog, and bug, with samples as-prepared, and after up to 3000 h of xenon arc exposure. The self-cleaning performances of the UVH coatings will be evaluated using two metrics and compared to uncoated substrates. The possible degradation mechanism of static water contact angle (sWCA) with weathering time in Weather-Ometer® chamber will be discussed. Surface morphology of the samples was examined by atomic force microscopy (AFM) to understand the surface change. The study indicates the benefit of the UVH coatings’ self-cleaning performance for autonomous sensors.

## Materials and methods

Borosilicate glass and hard coat-coated polycarbonate (PC) were pre-treated with air plasma treatment for 15 min using an ATTO™ plasma treater (Diener Electronics, Germany). The proprietary UVH coatings from PPG Industries, Inc. were spray-coated on pre-treated substrates by an ultrasonic spray process using an ultrasonic spray system Prism Ultra-Coat (Ultrasonic Systems, Inc., Haverhill, MA). The samples were then cured at 100 °C for 10 to 15 min. After curing, sWCA was measured by depositing three 2.0 µL droplets of deionized water on the surface of the sample and the average sWCA was calculated using ADVANCE software using a Kruss Drop Shape Analyzer DSA100 Instrument. Due to the hydrophobic nature of the UVH coatings, the sWCA was measured as 115° ± 1°. The surface free energy (SFE) was calculated with the Owens, Wendt, Rabel and Kaelble (OWRK) method using deionized water and diiodomethane contact angles. Some of the samples were then weathered for 500, 1000, 1500, 2000, 2500, and 3000 h in an ATLAS Ci5000 Weather–Ometer® (Atlas Material Testing Technology, Mount Prospect, Illinois, USA) using ASTM D7869, with several cycles of alternated xenon light exposure and dark environments, varying temperatures from 40 to 50 °C, and varying relative humidities from 50 to 95%. sWCA was measured and recorded from each set of samples. These samples were also analyzed by K-Alpha X-Ray Photoelectron Spectroscopy (XPS) with a base pressure better than 5 × 10^−9^ mbar to correlate the coating composition with the change of sWCA. AFM analyses were conducted using a Bruker Dimension Icon using an RTESPA-150 probe with 1 µm scan. A stiffer probe with 6N/m spring constant was used for glass when electrostatic were higher. The coating samples were also subjected to the below testbed using lab-simulated weathering conditions to evaluate their self-cleaning performances.

**Figure 2:**
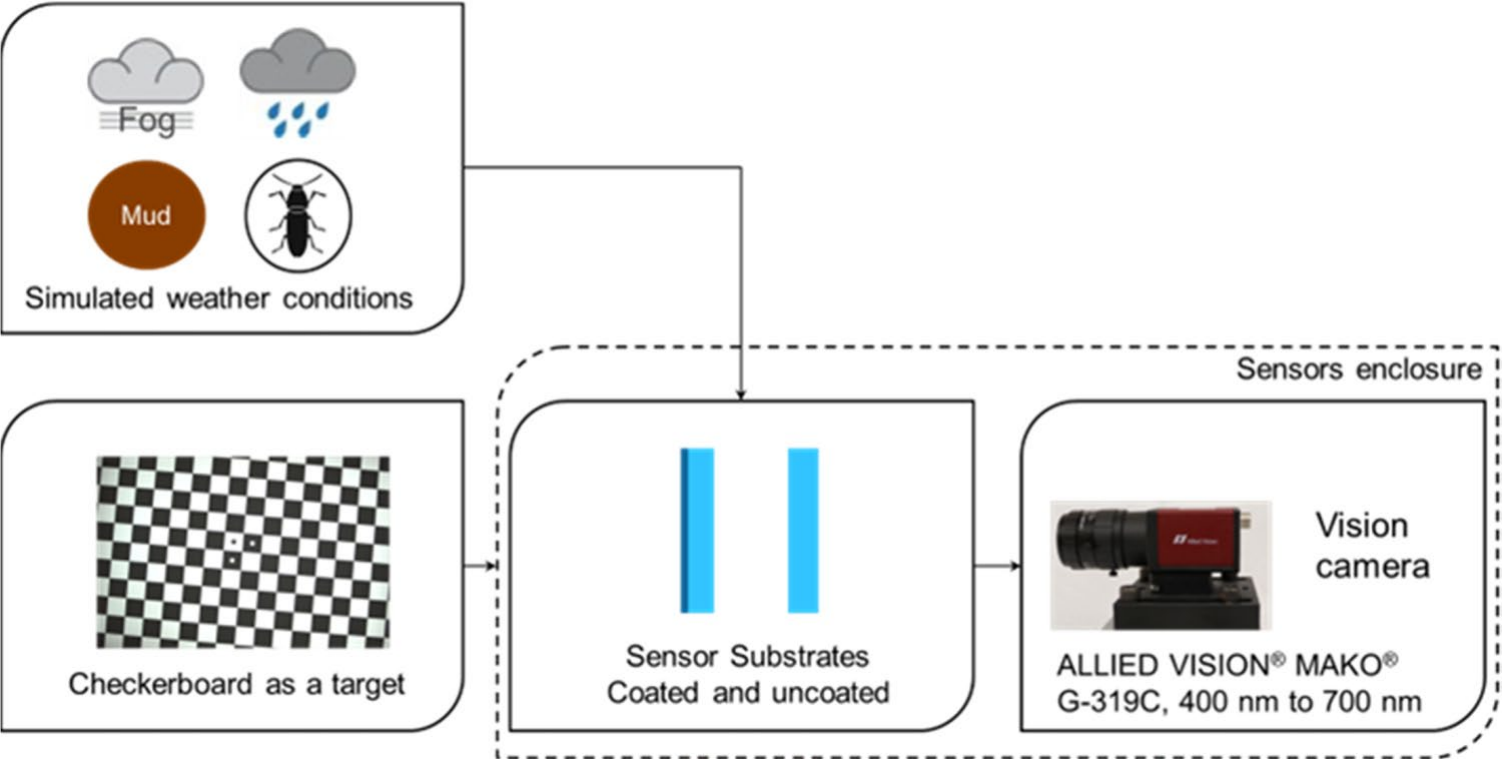
A block diagram showing a lab sensor testbed with lab-simulated weathering conditions.

A block diagram of a lab testbed setup is illustrated in Fig. 2. The testbed has a sensor enclosure hosting an ALLIED VISION® MAKO® G-319C vision camera with wavelength ranging from 400 to 700 nm. The camera is mounted on a small rail inside the water-tight enclosure. A checkerboard is used as a target for the vision camera. In the front of the sensor enclosure, there is a switchable opening for different sensor substrates (coated or uncoated) with a size of 6.35 × 12.7 cm. Four different weathering conditions, including rain, mud, bug, and fog, are applied to sensor substrates to impact the checkerboard images taken from the vision camera. A Digital Subscriber Line (DSL) camera is used to take images on the sensor substrate surface for visualization purposes. A control computer is utilized to control and record data from sensors.

A set of lab-simulated weathering conditions with contaminants, such as water, dirt/dust, and organic material, was established in the sensor testbed as shown in Table 1.

**Table 1:** Lab-simulated weathering conditions in the sensor testbed.

Water	Dirt/dust	Organic materials
Rain	Light mud	Bug (cricket)
Fog		

Rain was simulated in the lab with a pressurized vessel to feed deionized water to a nozzle controlled by a manual air control valve. The applied pressure, nozzle size, and angle determined the rain droplet size and vertical/horizontal velocities of the falling rain droplets. The nozzle was fixed at an angle of 30° of normal to the substrate to have a vertical velocity and horizontal velocity when falling to mimic a moving vehicle. The light rain was simulated with an average rain droplet size of around 0.5 mm, a vertical velocity of less than 2 m/s, and an estimated horizontal velocity of 3.3 m/s. The heavy rain was simulated with an average rain droplet size of around 1.8 mm, a vertical velocity of 6.6 m/s, and an estimated horizontal velocity of 11.4 m/s. A light mud condition was simulated by feeding a mixture of low-concentration salt (sodium chloride) and ISO ultrafine Arizona test dust (Powder Technology, Inc., MN) in deionized water in a pressurized vessel to a nozzle from which the mud solution was sprayed onto the sample surface. The light mud has a vertical velocity of 2.9 m/s and a horizontal velocity of 5.0 m/s. The lab-simulated fog was generated using an SPT SU-2081B digital ultrasonic humidifier filled with deionized water. The fog droplet diameters were less than 25 μm, with the maximum volume weighted fraction at 6 μm, which is corresponding to the fog size in nature^17^. A 2-feet long polyvinyl chloride (PVC) plastic tube directed the fog droplets from the humidifier to the surface of the testing sample at 55° from normal. A cricket from Zoo Med Can O' Mini Size Crickets was shot from a lab-made gun barrel with a press-release trigger and a compressed air source. The pressure was optimized so that the cricket can land on similar spots with consistency and at a similar speed of 45 miles per hour (mph) to simulate the highway driving condition.

After light mud tests, the haze was measured using a Color i7 Benchtop Spectrophotometer (X-Rite, Inc., Grand Rapids, MI) on samples after 0 h, 500 h, and 1000 h in Weather-Ometer® chamber. The samples were dried after mud spray before haze measurements.

Two metrics were used for camera image quality analyses. The signal-to-noise ratio (SNR) is a metric to measure image quality based on definitions by Gonzalez and Woods^18^. The signal from a test image t(x,y) after weathering is compared to a reference image r(x,y) before weathering. In this study, the reference image represents the original test chart image without weathering, and the test images show simulated weathering effects on the camera lens. SNR highlights the impact on the lens and its implications for overall imaging system quality, indicating resistance to weathering in driving scenarios for improved visibility and potential safety enhancements. The SNR is calculated using Eq. (1)^19^. A higher SNR value indicates better image quality and fewer weathering effects on the camera lens.

1 $$SNR = 10\,\log_{10} \left[ {\tfrac{{\Sigma_{0}^{{n_{x - 1} }} \Sigma_{0}^{{n_{y - 1} }} [r(x,y)]^{2} }}{{\Sigma_{0}^{{n_{x - 1} }} \Sigma_{0}^{{n_{y - 1} }} [r(x,y) - t(x,y)]^{2} }}} \right]$$

The second metric for image quality, particularly for sharpness, is modulation transfer function (MTF)^20^. Imatest explained the MTF as spatial frequency response (SFR). The equation of MTF is derived from the sine pattern contract at spatial frequency f. It is an image sharpness metric describing the spatial resolution of the imaging system as a whole (sensor, optics, electronics, any processing, etc.) at different frequency responses. Further, a modulation transfer function 50 (MTF50) is the spatial frequency where MTF is 50% of the low (0) frequency MTF (when the spatial frequency is 0, its MTF = 1). To compare the quality of the weathered image a better metric is the loss of MTF50 after weathering as compared to before weathering. In this metric, a lower value indicates a minimal reduction in image sharpness.

2 $$MTF50_{loss} = MTF50_{initial} - MTF50_{after}$$

Where MTF50_initial_ is the MTF50 before weathering, and MTF50_after_ is MTF50 after weathering. The MTF50_loss_ is the difference between MTF50_initial_ and MTF50_after_.

In this study, the MTF50_loss_, a measure of spatial resolution degradation, is developed to show the impact of weathering on the imaging system, particularly the effects of droplets or debris on the lens. The MTF50_loss_ revealed the adverse weather conditions' influence on the imaging system's ability to resolve fine details, including road signs or obstacles. Understanding these weathering effects is crucial for assessing the potential implications on the safety and efficiency of autonomous vehicles, as they rely heavily on precise imaging for navigation and decision-making.

The checkerboard is utilized for Modulation Transfer Function (MTF) or sharpness measurement because it can generate 346 Regions of Interest (ROIs), each containing an edge between black and white regions across the test chart image, resulting in a highly detailed sharpness (MTF50) map. The chart also includes three dots representing the image center, aiding in precise chart and camera system positioning.

MTF results for purely vertical or horizontal edges are highly sensitive to the relationship between the edge and pixel locations. To mitigate this sensitivity, edges are typically tilted by more than 2 or 3°, preventing potential issues. In the test chart setup, edge angles are set to a 5-degree inclination from vertical (V) or horizontal (H).

**Figure 3:**
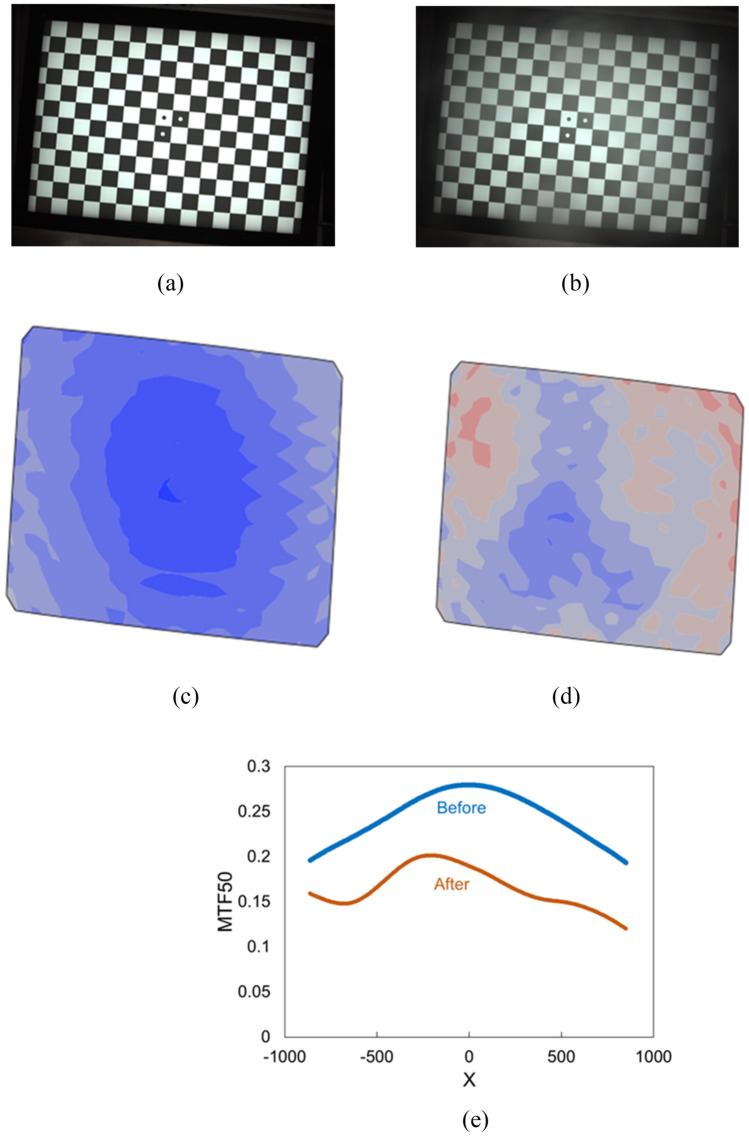
Checkerboard images and their MTF50 plots through glass substrate without UVH coatings taken by the vision camera before and after light rain. (**a**) checker board image before light rain, (**b**) checkerboard image after light rain, (**c**) MTF50 contour plot of (**a**), (**d**) MTF50 contour plot of (**b**), (**e**) MTF50 2D plot from (**c**) and (**d**). The calculated MTF50_loss_ is 0.085 from (**e**).

For example, Fig. 3a is the clear image of the checkerboard taken by the vision camera before light rain, and Fig. 3b is the blurry image after light rain, both through an uncoated borosilicate glass substrate without the UVH coatings. According to Eq. (1), the SNR is calculated as 10.53. Figure 3c is the corresponding 3D contour plot of MTF50 from Fig. 3a before light rain, and Fig. 3d is the corresponding 3D contour plot of MTF50 from Fig. 3b after light rain. By converting the 3D contour plots to 2D curves in Fig. 3e, MTF50_loss_ can then be calculated according to Eq. (2) as 0.085.

## Results

### sWCA and its possible degradation mechanism during weathering

**Figure 4:**
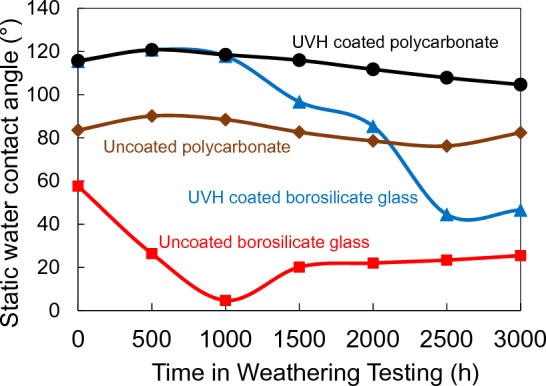
sWCA of UVH coatings-coated and uncoated borosilicate glass and polycarbonate substrates before and after different hours in xenon arc exposure tests.

Figure 4 shows the sWCA of UVH coatings-coated and uncoated borosilicate glass and hard coat-coated polycarbonate substrates before and after 500, 1000, 1500, 2000, 2500, and 3000 h in Weather–Ometer® chamber. The UVH coatings-coated polycarbonate substrate had the best results with a slight reduction of sWCA after 2000 h which was still higher than 100° even after 3000 h in Weather-Ometer® testing. The UVH coatings-coated borosilicate glass, however, started to degrade after 1500 h in Weather-Ometer® testing. After 2000 h in Weather-Ometer® testing, its sWCA was already below 90°. After 2500 h in Weather-Ometer® testing, its sWCA reduced to less than 50°. Basically, the low sWCA indicated that the UVH coatings had almost completely degraded from the borosilicate glass surface after 2000 h. Both results from borosilicate glass and hard coat-coated polycarbonate substrates indicated that the degradation didn’t happen for intermolecular bond but rather at the interface between UVH coatings and the substrate since the degradation of sWCA is substrate related. On the other hand, the uncoated borosilicate glass has very low sWCA from the beginning and went down further after Weather-Ometer® testing. The hard coat-coated polycarbonate substrate without UVH coatings maintained the sWCA around 80° to 90° throughout the testing.

**Figure 5:**
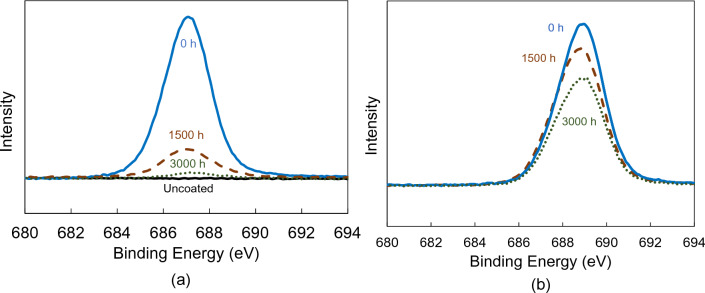
XPS F 1 s spectra of (**a**) UVH coatings-coated and uncoated borosilicate glass as-prepared (0 h), after 1500 and 3000 h after xenon arc exposure, (**b**) UVH coatings-coated polycarbonate as-prepared (0 h), after 1500 and 3000 h after xenon arc exposure.

An XPS analysis of several samples from Fig. 4 was carried out to understand the degradation of UVH coatings during the accelerated weathering. XPS fluorine (F) 1 s spectra in Fig. 5 indicated the rapid degradation of the F 1 s intensity of UVH coatings on borosilicate glass even after 1500 h after xenon arc exposure, compared with a relatively small drop of the F 1 s peak of UVH coatings on polycarbonate. After 3000 h, the F 1 s peak of UVH coatings on borosilicate glass is extremely weak, compared to still high F 1 s peak from UVH coatings on polycarbonate substrate. Due to the different chemical environments at the interface of UVH coatings on borosilicate glass and polycarbonate, there is a chemical shift of the F 1 s peak from a binding energy of 687.2 eV on borosilicate glass to 689.0 eV on a polycarbonate substrate.

**Figure 6:**
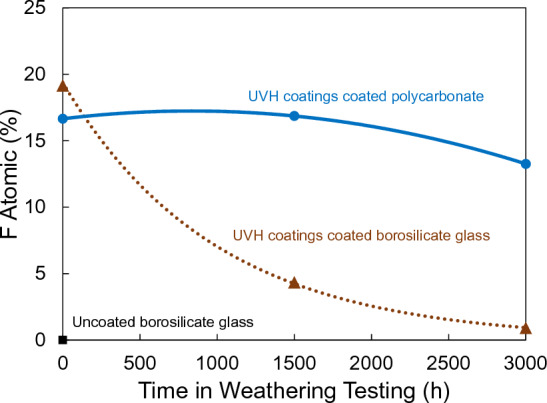
XPS results of F atomic percentage of UVH coatings-coated borosilicate glass and UVH coatings-coated polycarbonate without weathering, after 1500 and 3000 h weathering after xenon arc exposure vs uncoated borosilicate glass without weathering.

The plot of F 1 s atomic percentage vs hours in Weather-Ometer® chamber is illustrated in Fig. 6. The F 1 s atomic percentage of UVH coatings on borosilicate glass decreased rapidly, while the F 1 s atomic percentage of UVH coatings on polycarbonate decreased slowly.

### Surface morphology

**Figure 7:**
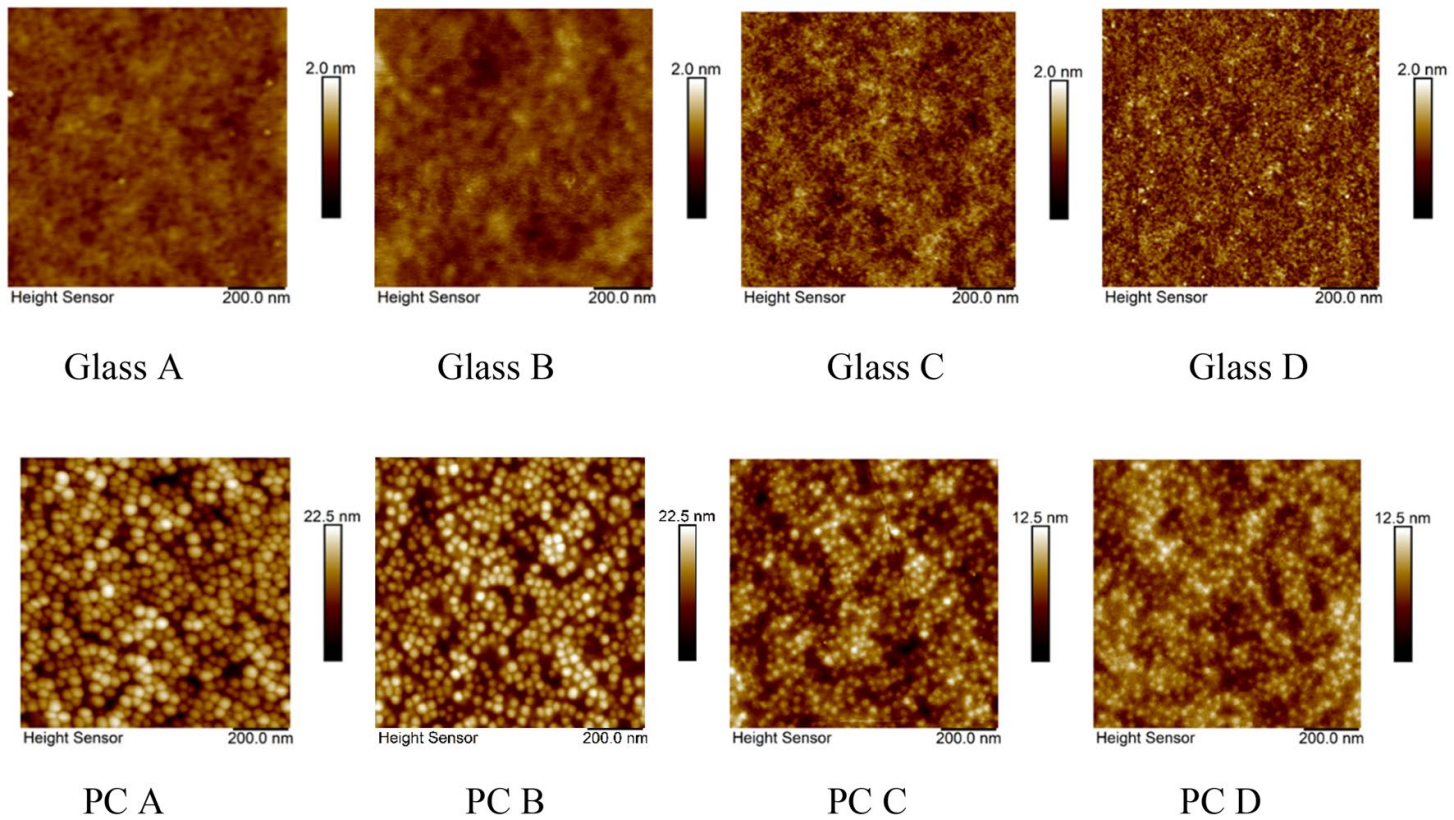
AFM images of samples on glass and PC substrates. Samples IDs are described in Table 2.

AFM analyses were carried out on uncoated surface vs three types of surfaces with UVH coatings (Table 2 and Fig. 7**)**. The AFM results indicate that uncoated glass samples are smoother than hard coat coated PC samples. After UVH coatings were coated on the surface, the surface became slightly rougher. However, after 2500 h in the Weather-Ometer® chamber, the surface roughness values on glass are very similar, while the surface roughness on PC became smaller. This is possibly explained that the weathering action from the Weather–Ometer® chamber smoothened out the surface of UVH coatings coated PC substrate with a hard coat.

**Table 2:** AFM sample ID and description, and their Ra and RMS.

Sample ID	Sample description	Time in weather-ometer® chamber (h)	Weathering in lab testbed	Ra (nm)	RMS (nm)
Glass A	Uncoated glass	0	no	0.08	0.10
Glass B	UVH on glass	0	no	0.14	0.14
Glass C	UVH on glass	2500	no	0.15	0.14
Glass D	UVH on glass	2500	yes	0.16	0.20
PC A	Hard coat coated PC	0	no	2.62	3.31
PC B	UVH on hard coat coated PC	0	no	2.89	3.43
PC C	UVH on hard coat coated PC	2500	no	1.32	1.64
PC D	UVH on hard coat coated PC	2500	yes	1.13	1.40

### Self-cleaning performances under inclement weather

The self-cleaning performances of UVH coatings vs those of uncoated samples were evaluated by comparing the front images of the samples taken by the DSL camera before and after weathering, and the images of the checkerboard taken by the ALLIED vision camera through the substrates before and after weathering. The SNR and MTF50_loss_ were then calculated from the images of the checkerboard. The results are presented in below sections with light rain, light mud, fog, and bug weathering.

#### Light rain

**Figure 8:**
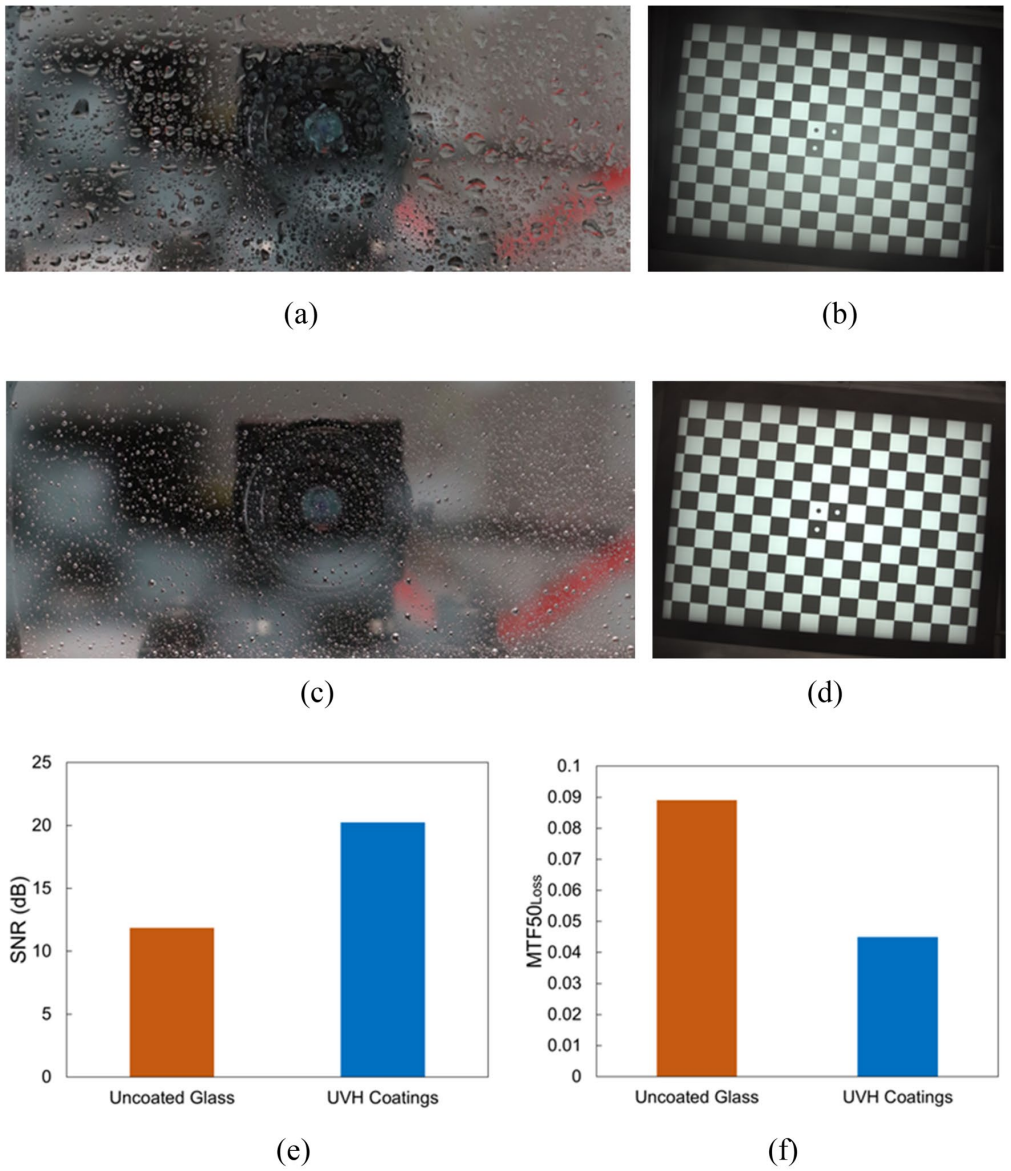
Vision camera performance after lab-simulated light rain with uncoated glass and UVH coatings-coated glass after 500 h in Weather-Ometer® chamber. (**a**) the front image of uncoated glass with light rain droplets, (**b**) the checkerboard image through uncoated substrate from the vision camera, (**c**) the front image of UVH coatings-coated glass with light rain droplets, (**d**) the checkerboard image through UVH coatings-coated substrate from vision camera, (**e**) SNR, and (**f**) MTF50_loss_.

Figure 8a is the front image of the uncoated glass after light rain application. The droplets on the substrate surface are larger than those in the front image (Fig. 8c) of the UVH coatings-coated glass after light rain applications. The checkerboard image in Fig. 8b through the uncoated glass is blurry compared to the clear checkerboard image in Fig. 8d through the UVH coatings-coated glass. The SNR of the UVH coatings-coated sample is much higher than the SNR of the uncoated glass (Fig. 8e). Additionally, the MTF50_loss_ of UVH coatings-coated sample is much lower than the MTF50_loss_ of the uncoated glass.

#### Light mud

**Figure 9:**
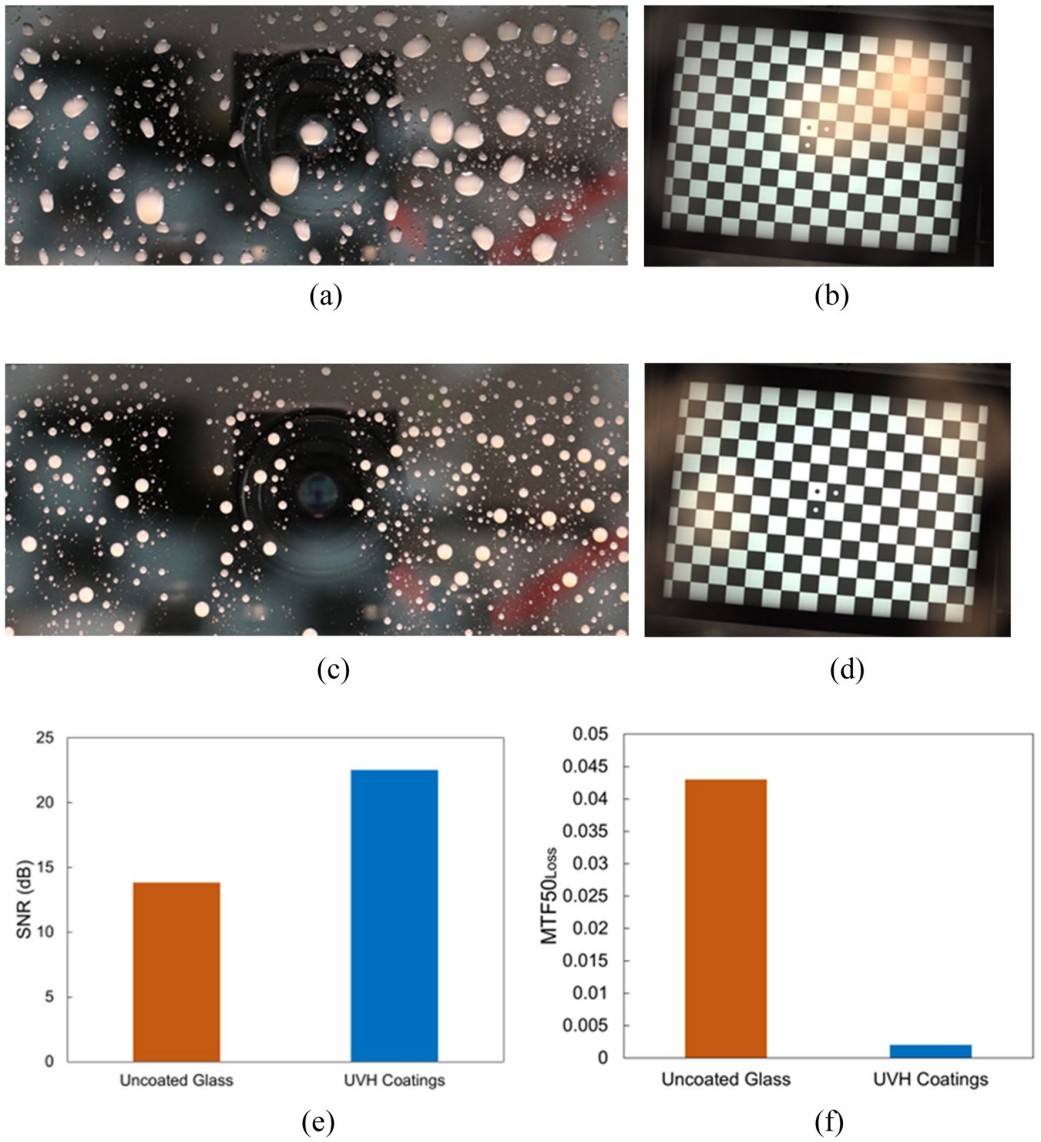
Vision camera performance after lab-simulated light mud with uncoated glass and UVH coatings-coated glass. Both samples had 0 h xenon arc exposure. (**a**) the front image of uncoated glass with light mud droplets while still wet, (**b**) the checkerboard image through uncoated substrate from vision camera, (**c**) the front image of UVH coatings-coated glass with light mud droplets, (**d**) the checkerboard image through UVH coatings-coated substrate from vision camera, (**e**) SNR, and (**f**) MTF50_loss_.

Figure 9a is the front image of the uncoated glass after light mud application. The muddy droplets on the substrate surface are larger than those in the front image (Fig. 9c) of the UVH coatings-coated glass after light mud applications. The checkerboard image in Fig. 9b through the uncoated glass has a large blurry area, probably corresponding to a large muddy droplet on the substrate surface, compared to the clear checkerboard image in Fig. 9d through the UVH coatings-coated glass. The SNR of UVH coatings-coated sample is higher than the SNR of the uncoated glass (Fig. 9e). Again, the MTF50_loss_ of UVH coatings-coated sample is much lower than the MTF50_loss_ of the uncoated glass.

Haze value was measured for UVH coatings samples and uncoated glass samples after light mud spray and after dry to affirm self-cleaning behavior of UVH coatings. UVH coatings after 0 h, 500 h, and 1000 h in the Weather-Ometer® chamber show almost no haze after light mud spray. This is because after droplet dried, there left only very small amount of dispersed and dried mud on the surface while most of them fell off. On the other hand, the uncoated glass samples after 0 h, 500 h, and 1000 h in the Weather-Ometer® chamber show high haze after light mud spray and dry, especially after 500 h and 1000 h in the Weather-Ometer® chamber (Table 3). The results indicate that the hydrophobic surface with sWCA above 115° can prevent dirt accumulation on its surface, while hydrophilic surface with sWCA below 60°, especially with the super-hydrophilic surface (sWCA ≤ 5°) can accumulate more dirt, probably because of the sheeting effect of the hydrophilic surface.

**Table 3:** Haze of UVH coatings and uncoated glass samples after light mud spray.

Sample	Hours in Weather-Ometer®	sWCA (°)	Haze (%) after light mud spray
UVH coatings	0	115.39	0
UVH coatings	500	120.78	0
UVH coatings	1000	117.87	0.07
Uncoated glass	0	57.66	1.1
Uncoated glass	500	26.41	8.18
Uncoated glass	1000	4.73	19.87

#### Fog

**Figure 10:**
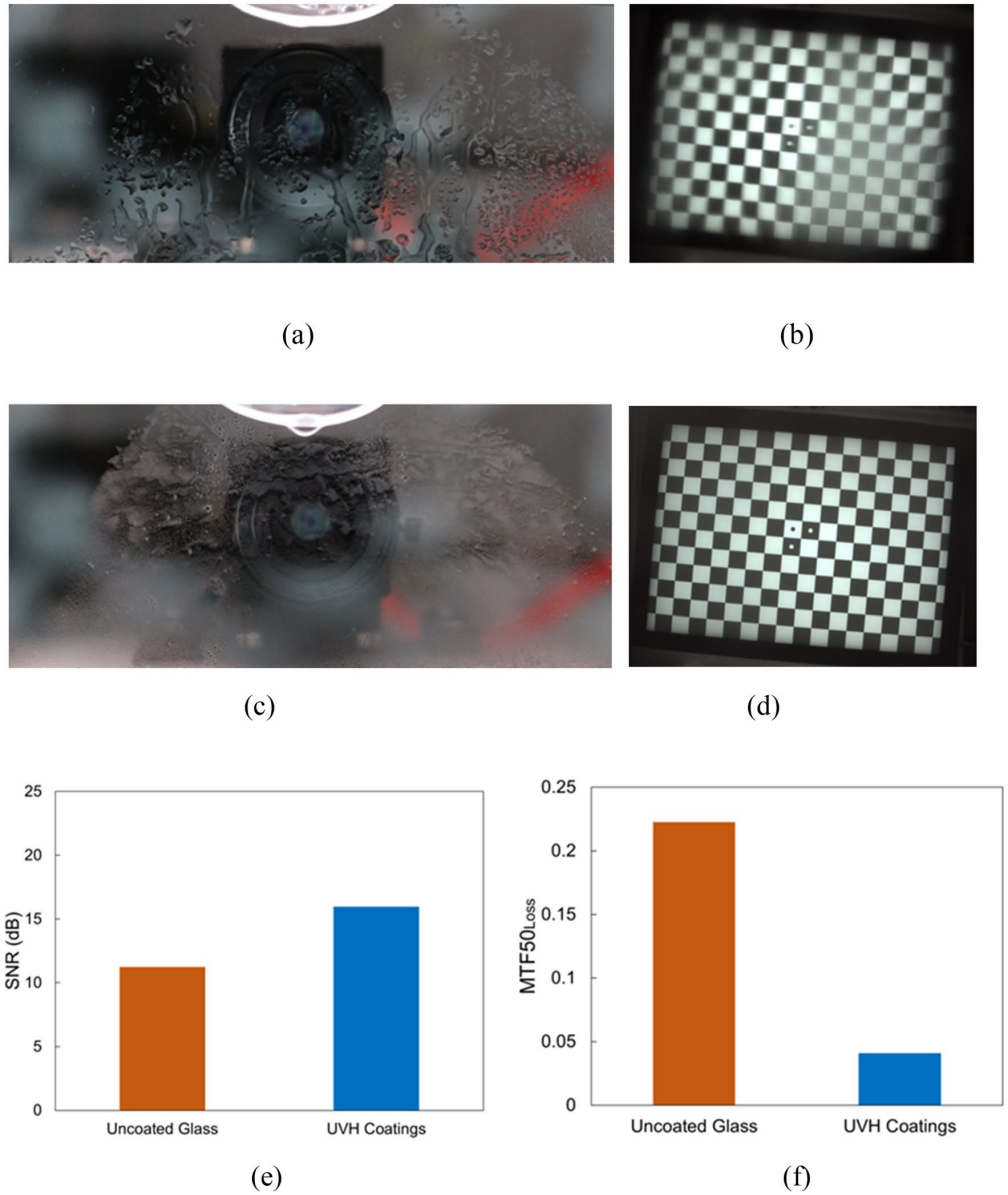
Vision camera performance after lab-simulated fog with uncoated glass and UVH coatings-coated glass after 1000 h in a Weather-Ometer® chamber. (**a**) the front image of uncoated glass with fog, (**b**) the checkerboard image through the uncoated substrate from the vision camera, (**c**) the front image of UVH coatings-coated glass with fog, (**d**) the checkerboard image through UVH coatings-coated substrate from the vision camera, (**e**) SNR, and (**f**) MTF50_loss_.

Figure 10a is the front image of the uncoated glass after fog weathering. The fog formed large and irregular water puddles on the uncoated substrate surface. On the other hand, the fog formation on UVH coatings-coated glass is denser with possible smaller particles (Fig. 10c). The checkerboard image in Fig. 10b through the uncoated glass has blurry areas, probably corresponding to large water puddles on the substrate surface, compared to the clear checkerboard image in Fig. 10d through the UVH coatings-coated glass. The SNR of UVH coatings-coated sample is higher than the SNR of the uncoated glass (Fig. 10e). As in the previous two sections, the MTF50_loss_ of UVH coatings-coated sample is much lower than the MTF50_loss_ of the uncoated glass.

#### Bug

**Figure 11:**
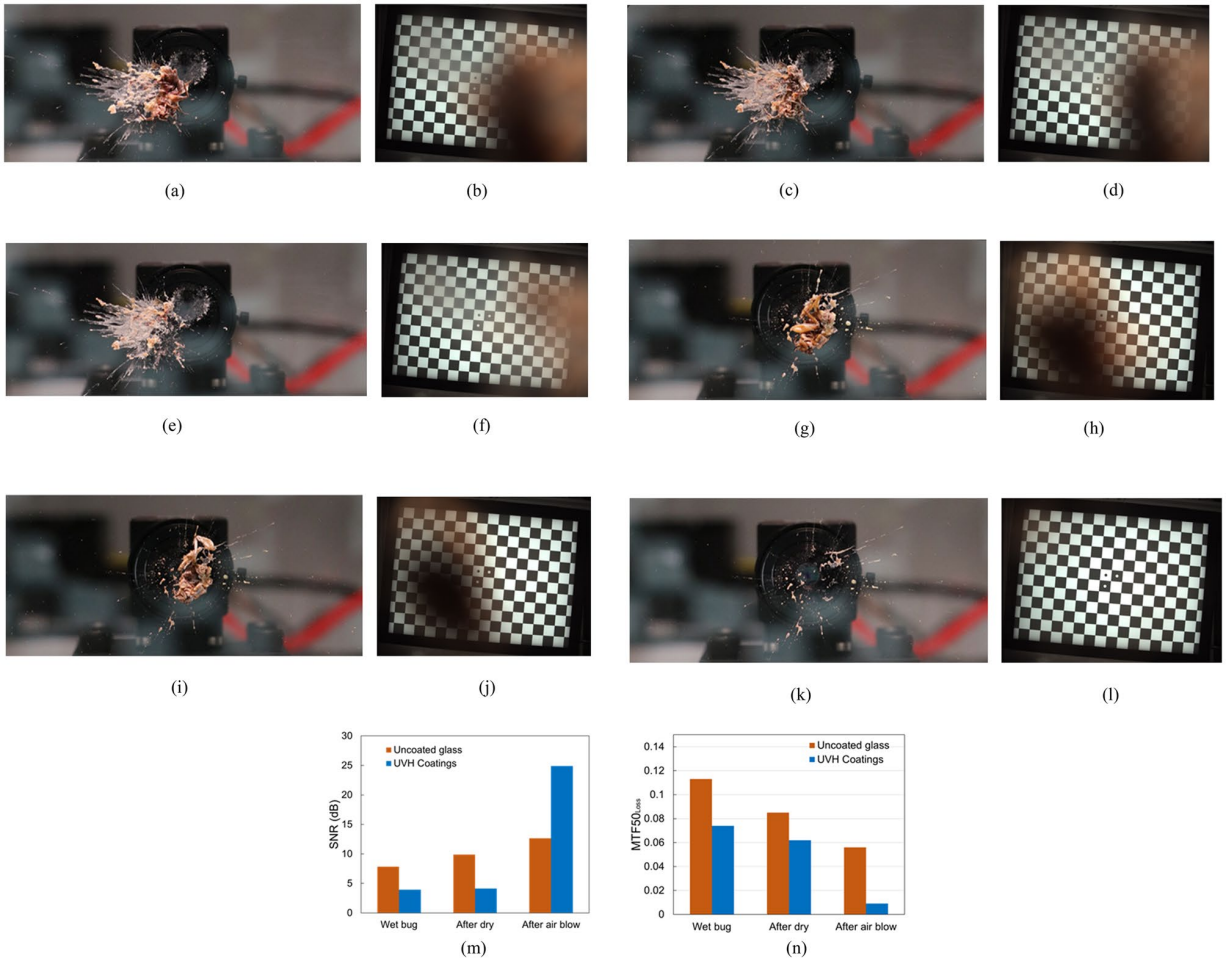
Vision camera performance after lab-simulated bug testing on uncoated glass and UVH coatings-coated glass after 500 h in a Weather-Ometer® chamber. (**a**) The front image of uncoated glass with wet bug after bug application, (**b**) the checkerboard image of the wet bug through uncoated substrate from vision camera, (**c**) the front image of bug after dry on uncoated glass, (**d**) the checkerboard image of the bug after dry through uncoated glass from vision camera, (**e**) the front image of bug after air blow on uncoated glass, (**f**) the checkerboard image of the bug after air blow through uncoated glass from vision camera, (**g**) the front image of UVH coatings-coated glass with wet bug after bug application, (**h**) the checkerboard image of the wet bug through UVH coatings-coated glass from vision camera, (**i**) the front image of bug after dry on UVH coatings-coated glass, (**j**) the checkerboard image of the bug after dry through UVH coatings-coated glass from vision camera, (**k**) the front image of bug after air blow on UVH coatings-coated glass, (**l**) the checkerboard image of the bug after air blow through UVH coatings-coated glass from vision camera, (**m**) SNR, and (**n**) MTF50_loss_.

After bug application, three different stages were studied, including wet bug, bug after dry, and after air blow on both uncoated glass and UVH coatings-coated glass. Figure 11a,c,e are the front images of the bug on the uncoated glass when wet, after dry, and after air blow, and Fig. 11b,d,f) are the corresponding checkerboard images through the uncoated glass from the vision camera. As one can see, on the uncoated glass, the checkerboard image is slightly better after dry, and much better after air blow. However, there is still a blurry area in Fig. 11f since the bug is still on the surface after air blow on the uncoated glass. Figure 11g,i,k are front images of the bug on UVH coatings-coated glass when wet, after dry, and after air blow, and Fig. 11h,j,l are corresponding checkerboard images through the UVH coatings-coated glass from the vision camera. Therefore, on the UVH coatings-coated glass, the checkerboard image is slightly better after dry, and is clear after air blow. This is because the bug is almost gone after air blow, with only some residuals left on the hydrophobic surface. This phenomenon has also been observed by Krishnan et al. that the insect residue area was found to correlate well with the wettability properties of the surface, i.e. the hydrophobic surfaces performed better than the hydrophilic surface during insect’s impacts^21^. The SNR for the wet bug and after dry is higher for uncoated glass than that for UVH coatings because the bug is slightly off-center on uncoated glass. This probably increased the SNR for uncoated glass as shown in Fig. 11b,d,f that the right-side image is clear. Nevertheless, after air blow, the SNR is higher for UVH coating-coated glass than that for uncoated glass. On the other hand, the MTF50_loss_ is much lower for UVH coating-coated glass than that for uncoated glass for all cases of wet bug, after dry, and after air blow.

## Discussion

Both self-cleaning performances and UV durability are extremely important features for autonomous sensors, such as cameras, to ensure accurate signal reading. Understanding the mechanisms behind the self-cleaning performances and UV durability of hydrophobic coatings is critical for driving further improvements in these areas.

The hydrophobic state is a surface phenomenon with low surface energy and high water contact angle. An uncoated surface, such as glass, usually is in the hydrophilic state for which the static water contact angle is lower than 90°, depending on how clean or dirty the uncoated glass surface is. For example, a UVH coating surface is measured with its sWCA at 115° ± 1° and a surface energy of 12.65 ± 0.39 mN/M, and an uncoated glass substrate is measured with its sWCA at 40 ± 10°, and a surface energy of 51.39 ± 0.71 mN/M of samples without xenon arc exposure. The sWCA of UVH coatings is only around 115° ± 1° even though the surface energy is as low as 12.65 ± 0.39 mN/M because of the smooth surface with Ra in the range of 0.14 nm on glass substrate to 3.54 nm on hard coat coated PC. From RMS and Ra values, the UVH coatings coated samples have slightly higher values than uncoated surface (Table 2).

**Figure 12:**
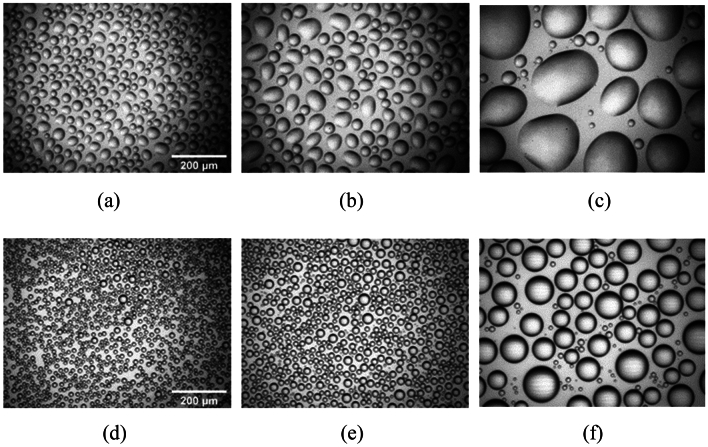
Fog formation on UVH coatings vs uncoated glass after different times of continuous fog impact, (**a**) 1 min on uncoated glass, (**b**) 2 min on uncoated glass, (**c**) 10 min on uncoated glass, (**d**) 1 min on UVH coatings, (**e**) 2 min on UVH coatings, and (**f**) 10 min on UVH coatings.

Due to its hydrophobic state of the UVH coatings, water will bead up on its surface with a high contact angle. On the other hand, water droplets will more likely form large puddles on the uncoated glass due to low water contact angle. Figure 12 is a set of images of water droplets on UVH coatings and uncoated glass after 1 min, 2 min, and 10 min of continuous fog impact. As one can see, after 10 min, the water droplets on the uncoated glass (Fig. 12c) are much larger than the droplets on UVH coatings (Fig. 12f). Due to these large water droplets, or oval-shaped water puddles to be exact, the image taken by the vision camera through the glass sample with these puddles will have significant optical distortion, leading to low SNR and high MTF50_loss_. On the UVH coating surface, the water droplets are smaller and round in shape, which will have less optical distortion on image quality taken by the vision camera, hence with high SNR and low MTF50_loss_. Another factor to affect the image quality is the water droplet coverage area on the substrate surface. The lower the water coverage area the less image distortion. Using ImageJ software calculation, the droplet coverage area is 67.3% on uncoated glass, and 54.1% on UVH coatings surface after 10 min of fog application. The less droplet coverage on UVH coatings also contributed to better image quality with higher SNR and lower MTF50_loss_.

**Figure 13:**
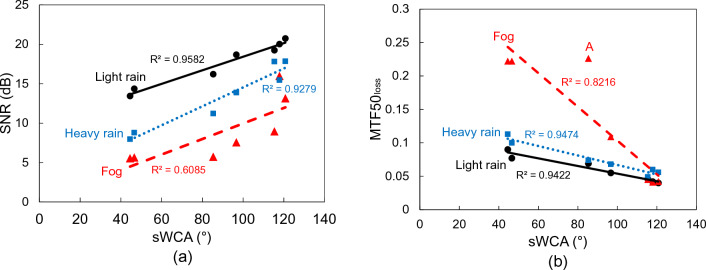
The correlations of SNR and MTF50_loss_ with sWCA after light rain, heavy rain, and fog impacts, (**a**) between SNR and sWCA, and (**b**) between MTF50_loss_ and sWCA. UVH coatings-coated glass samples were subjected to weathering for 0, 500, 1000, 1500, 2000, 2500, and 3000 h in a Weather-Ometer® chamber.

Figure 13a shows the correlation between SNR and sWCA for UVH coatings samples weathered in a Weather-Ometer® chamber for 0, 500, 1000, 1500, 200, 2500, and 3000 h, and Fig. 13b is the corresponding correlation between MTF50_loss_ and sWCA. As one can see, regardless of weathering hours in a Weather-Ometer® chamber, both SNR and MTF50_loss_ have strong linear relationships with sWCA in the case of light rain and heavy rain, with SNR and sWCA having a positive correlation, and MTF50_loss_ and sWCA having a negative correlation. However, in the case of fog, the SNR has a poor linear correlation with sWCA, and there is a significant outlier point A for MTF50_loss_ vs sWCA correlation. This may be explained by the effect of relative humidity in the lab during the testbed measurements in the span of a few months. The relative humidity was not controlled in the lab. Due to the small droplet size of around 6 μm with a maximum size of 25 μm, the effect of relative humidity on the evaporation of fog droplets will be much higher than on the evaporation of rain droplets, which are in the range of 0.5 mm size for light rain and 2 mm size for heavy rain. Thus, the evaporation of water from rain droplets may be negligent during the experiments, resulting in accurate data acquisition. The linear correlations with a R^2^ value of > 0.92 between SNR and sWCA, and MTF50_loss_ and sWCA of light rain and heavy rain indicate that the sWCA is a good indicator for vision camera image quality and accurate signal reading. This may also lead to the conclusion that a hydrophobic coating is better for self-cleaning performances for safe autonomous driving.

To maintain the self-cleaning performances over time when the sensors are outdoors, it is critical that the surface maintains its hydrophobicity. This means that it requires a slow decrease of sWCA with time. Xenon arc exposure test in a Weather–Ometer® chamber is the accelerated testing to understand the degradation of the performance over time under simulated cycling of heat, light, and moisture/water. The UVH coatings on hard coat-coated polycarbonate show a much slower decrease of sWCA over 3000 h in a Weather-Ometer® chamber than the UVH coatings directly coated on borosilicate glass. XPS results indicated that the possible degradation of hydrophobicity is the loss of fluorine, corresponding to the decrease of F 1 s peak (Figs. 5 and 6). Lisco et al. investigated the degradation of hydrophobic, anti-soiling coatings for solar module cover glass, revealing a significant decrease in the fluorine concentration at the surface of the coating, as shown in XPS F 1 s spectra, with approximately 34% loss after 500 h of UV exposure, and 57% overall loss after 2000 h of UV exposure^22^. It is suggested that the loss of fluorine correlated with the decrease in hydrophobicity. Tao et al. reported a degradation of tridecafluoro-1,1,2,2,tetrahydrooctyl trichlorosilane with significant decrease of CF_2_ XPS peak after 10 cycles of UV nanoimprints, indicating the loss of F atoms through UV exposure^23^. Peczonczyk et al. reported a similar drop of F 1 s peak intensity with hydrophobic coatings after weathering^13^. Therefore, to maintain a robust UV durability of hydrophobic coatings containing fluorine, it is essential to slow the degradation or loss of fluorine in the coatings. The degradation of UVH coatings is also observed from the AFM results on PC substrate, with both decreased surface roughness and decreased contrast in adhesion force image (Table 2). A UV-durable hydrophobic coating will ensure the longevity of its self-cleaning performance after an extended outdoor exposure of the sensor functional coatings.

**Figure 14:**
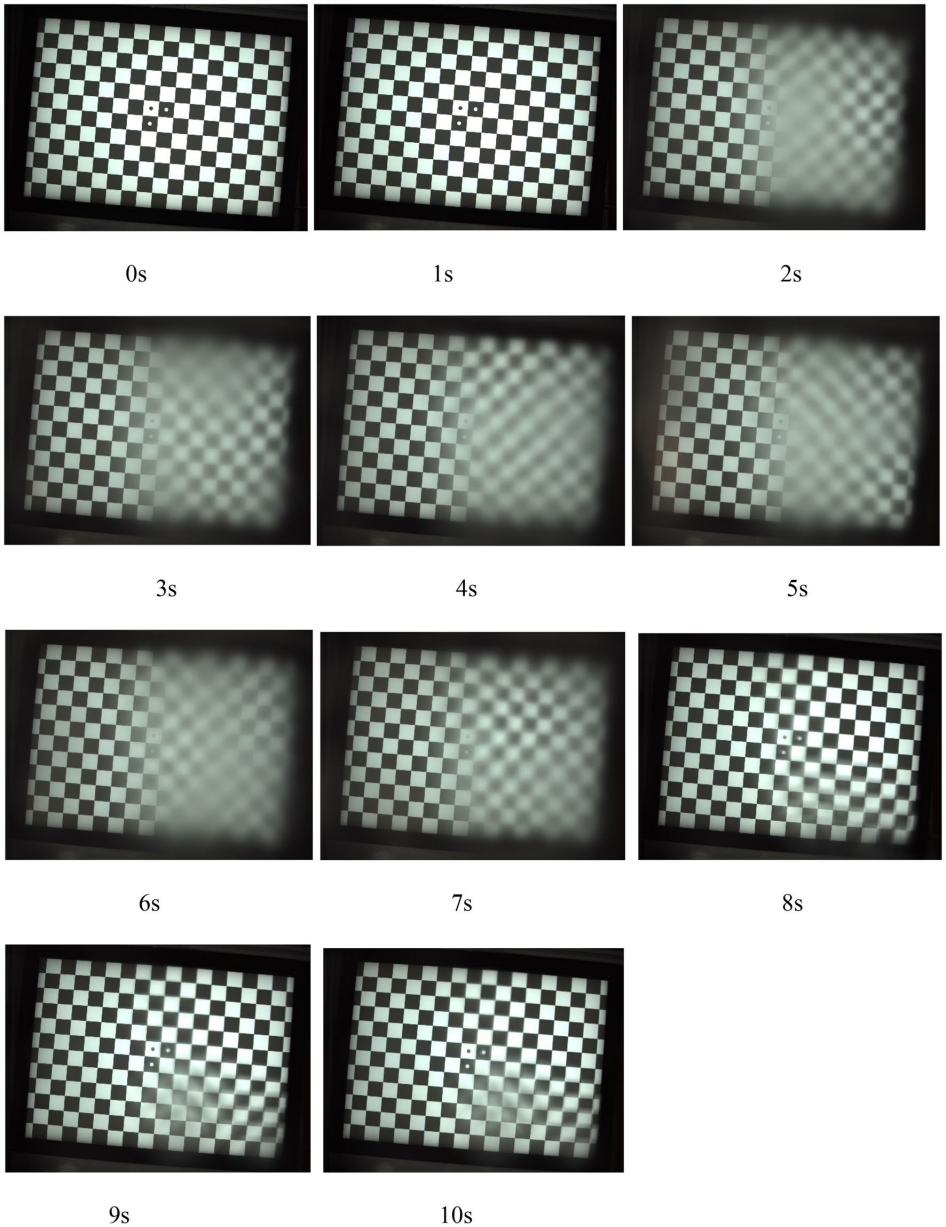
Continuous image frames (1 s to 10 s) from a video of heavy rain on a half-coated-half-uncoated sample before a vision camera to simulate a driving condition under heavy rain with a horizontal velocity of 11.4 m/s.

**Figure 15:**
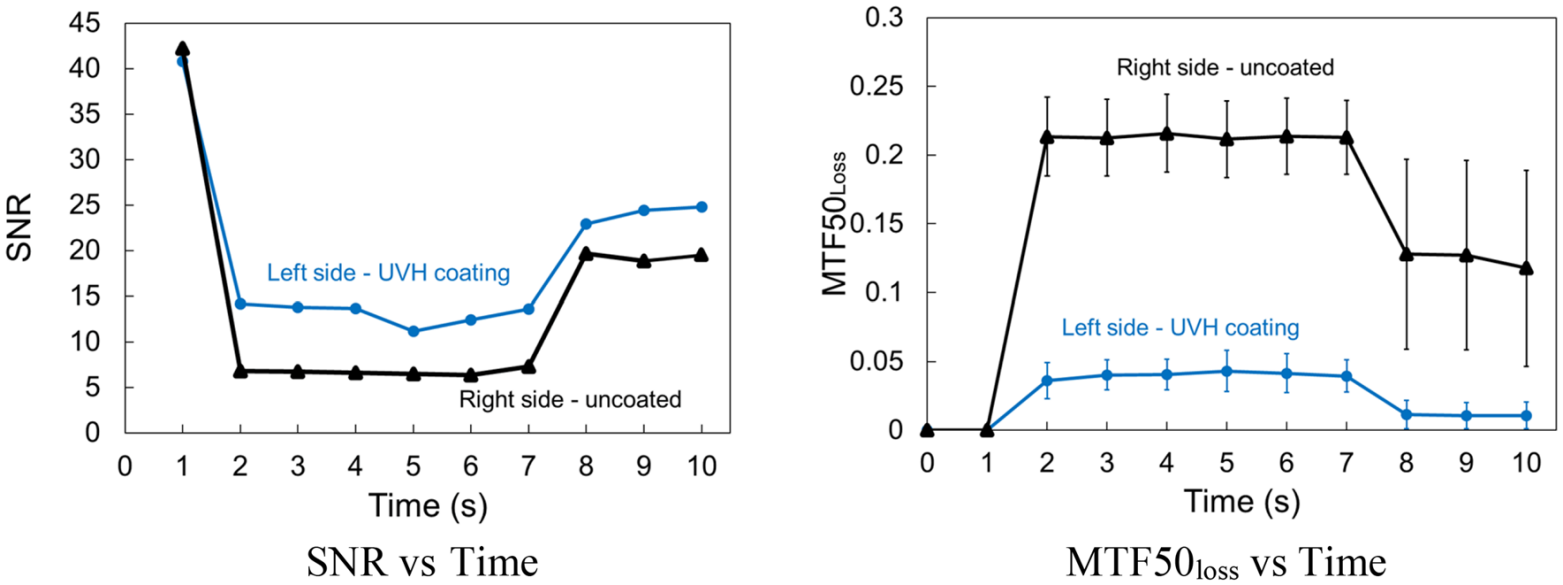
Calculated SNR and MTF50_loss_ values of left side with UVH coatings and uncoated right side from continuous image frames (1 s to 10 s) of Fig. 14.

To further understand the relationship between the calculated SNR and MFT50_loss_ and possible practical driving experience, a half-coated-half-uncoated sample was evaluated under heavy rain with an estimated horizontal velocity of 11.4 m/s. The image quality from the camera is what the computer will read information from the camera. A clear image means that the information from camera is accurate, and a very blur image shows that the information is distorted, which will lead the computer to make incorrect decision. Figure 14 is the continuous image frames (1 s to 10 s) from a video of heavy rain on a half-coated-half-uncoated sample before a vision camera to simulate a driving condition under heavy rain with an estimated horizontal velocity of 11.4 m/s. The heavy rain started after 1 s of the video, and stopped after 7 s of the video. The left side of the sample was coated with UVH coatings, and the right side of the sample was not coated. The images in Fig. 14 indicate that before rain started, there is no difference of image quality between left and right sides. After rain started, the left side is still clear with high image quality, while the right side is very blur. After rain stopped, left side is still clear, and the right side has the residual blurriness. This result corresponds both calculated SNR and MTF50_loss_ from the left and right sides of the images with the middle four columns were not counted for either left or right sides to avoid edge interference (Fig. 15). Initially the SNR value is the same from left to right. When rain started, SNR dropped for both sides, however, the coated left side is still higher than the uncoated right side. After rain stopped, SNR slightly increased for both sides with the left side still higher. A similar trend is observable in MTF50_loss_. Prior to the simulated rain, the overall image sharpness was 0.26, with both the left and right sides exhibiting comparable sharpness. As the rain started, the left side showed an MTF50_loss_ of only 0.04, indicating the maintenance of sharpness and precise recognition of black and white boundaries. On the other hand, the right side experienced an increase in MTF50_loss_ to 0.21, signifying a lack of sharpness and a loss of information on the black and white boundaries. After the rain stopped, the left side recovered to its initial state, with an MTF50_loss_ of 0.01. However, the right side, influenced by residue from the wafer film and droplets, showed an average MTF50_loss_ of 0.12 with significant variation. The sharpness measurements unveil the image quality throughout the rain conditions, showing that the UVH coating effectively kept the lens surface dry during heavy rain and preserved image sharpness. The results also show that the SNR and MTF50_loss_ values are more sensitive than human eyes, for example the SNR and MTF50_loss_ values of the left side for 0 s and 2 s are very different but human eye may see these two images very similar.

While current research focused on lab-simulated weathering conditions, a plan has been scoped to conduct a real-world weathering at Nevada Automotive Test Center (NATC). The results of this real-world weathering testing at NATC will be reported later.

## Conclusion

The UVH coatings have demonstrated their excellent self-cleaning performances and UV durability to enhance the accurate signal reading of autonomous sensors for safe driving. The evaluation results of UVH coatings vs uncoated glass for a vision camera under lab-simulated weathering conditions, such as light rain, light mud, fog, and bug, indicate the significant benefits of acquiring high-quality images through the UVH coatings, leading to high SNR and low MTF50_loss_. For the coating to maintain UV durability, thus prolonging the self-cleaning performance outdoors over time, it is essential to maintain hydrophobicity by reducing the loss of the fluorine concentration in the fluorine-containing coatings.

### Supplementary Information

Supplementary Video 1.

## Data Availability

All data generated or analyzed during this study are included in this published article and its supplementary information files.
